# Open questions on chemistry in the synthesis and characterization of superheavy elements

**DOI:** 10.1038/s42004-021-00529-8

**Published:** 2021-06-10

**Authors:** Patrick Steinegger

**Affiliations:** 1 Laboratory of Radiochemistry, Paul Scherrer Institute, Villigen PSI, Switzerland; 2 Laboratory of Inorganic Chemistry, Department of Chemistry and Applied Biosciences, ETH Zurich, Zürich, Switzerland

**Keywords:** Nuclear chemistry, Inorganic chemistry, Thermodynamics

## Abstract

Superheavy elements are ideal for furthering our understanding of relativistic effects and how they affect physicochemical properties of heavy elements. In this comment, the author discusses the role of chemistry in the synthesis of new elements before addressing the future challenges concerning the chemical characterization of superheavy elements.

In the quest of exploring the island of stability and the regions beyond^1^, scientist have regularly added new elements to the periodic table. Today’s approach for the synthesis of new chemical elements, the superheavy elements (SHEs) with atomic numbers *Z* ≥ 104, represents the alchemist’s long-sought dream come true: Using nuclear fusion-evaporation reactions, the production of gold from “base” elements would only be a matter of choosing the right combination of fusing elements. In this artificial production pathway, ion beams of typically lighter elements, the projectiles, are accelerated in large particle accelerators and guided onto thin targets consisting of heavier elements. If the energy of the incoming ions is large enough to overcome the repulsive Coulomb forces between the nuclei of projectile ions and target atoms, SHEs can be formed in complete fusion. However, strongly competing side reactions greatly diminish the probability of a successful synthesis and survival of such new elements. Therefore, experimentalists are usually left with single atoms per day, week or even month for their subsequent experiments.

Unlike in earlier days, the discovery of new chemical elements has evolved about 50 years ago from a discipline of chemistry to research conducted by physicists. The scarcity of available atoms per time, as well as their declining half-lives, has made unambiguous identification by chemical means increasingly demanding. Thus, the last chemical element discovered by chemists was dubnium (Db, *Z* = 105) in 1968, using gas-phase thermochromatography^2^. Besides seaborgium (Sg, *Z* = 106), all following discovery experiments employed large electromagnetic separators to isolate the synthesized SHEs from the projectile beam and the plethora of unwanted by-products. After having successfully traveled through the separator, the SHEs are implanted into a silicon detector, where their unique radioactive decay is recorded. This is how the 7^th^ period of the periodic table has recently been completed with the addition of four new members, namely nihonium (Nh, *Z* = 113), moscovium (Mc, *Z* = 115), tennessine (Ts, *Z* = 117), and oganesson (Og, *Z* = 118)^3,4^.

Meanwhile, radiochemistry experiments must go the extra mile: Between production and detection, chemists must introduce sophisticated rapid chemical systems that help us to learn more about the chemical behavior of said elements^5^. On the one hand, liquid-phase chemical investigations were conducted for generally more long-lived, lighter SHEs, namely rutherfordium (Rf, *Z* = 104), Db, and Sg as (oxo)halides in aqueous phase. At the same time, gas-adsorption chromatography experiments have largely prevailed across the entire range of SHEs. The latter offers the advantage of fast processing in combination with a comparably simple online detection scheme of short-lived radionuclides. One way or the other, studying the physicochemical properties of SHEs at the one-atom-at-a-time level helps us advance our understanding of relativistic effects. Having considerable impact on heavy elements in general, these effects complicate theoretical prediction of associated complex, physicochemical characteristics.

## Synthesis of new elements

**Fig. 1:**
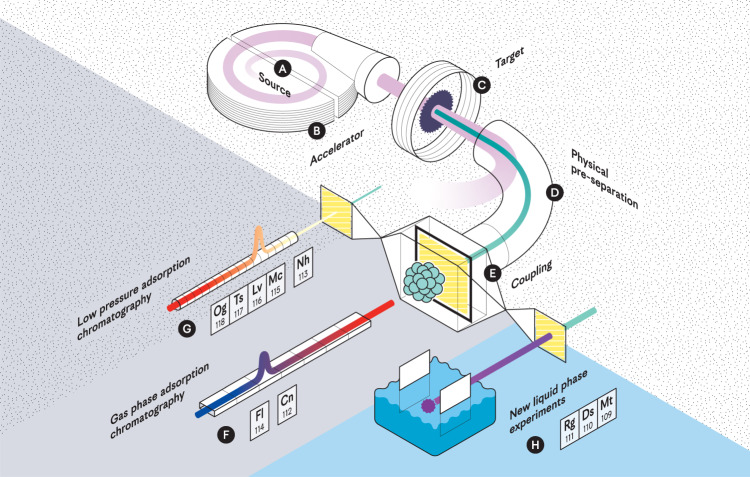
The impact of (radio)chemistry on the synthesis and characterization of superheavy elements. Pathway of production and chemical investigation with **A** the provision of the projectile element in a suitable chemical form for the introduction into the ion source, **B** the accelerator (shown for completeness), **C** the advancement of target technology for increasing heavy ion beam intensities, **D** the physical pre-separator (shown for completeness) providing superheavy elements at the one-atom-at-a-time level, **E** the coupling between the physics instrumentation and the chemistry experiments, **F** state-of-the-art gas phase adsorption chromatography with improved statistics, **G** low pressure adsorption chromatography for faster processing times in combination with higher stationary surface temperatures, and **H** entirely new liquid phase experiments with so far uncharacterized SHEs.

Aside from the constraints of physics, experimentalists today need two things in order to improve their odds for the successful synthesis of a new element: First, they need a high intensity heavy-ion beam and secondly, they need a heavy actinide target of high stability, which can cope with the provided ion beam intensities over month-long irradiation periods. For both parts, constant contributions of radio(chemists) remain vital during the careful processing of isotopically enriched, and thus rather expensive materials. Before the heavy ions can be accelerated, the starting material needs to be ionized. It must be provided in a chemically suitable form (see **A** in Fig. 1), allowing it to be steadily introduced into the ion source. For example in case of ^50^Ti, chemists devised two suitable forms, namely, metallic ^50^Ti to be sputtered from a Penning ion source^6^ as well as the evaporation of the comparably volatile, but complex organometallic compound Cp*Ti(CH_3_)_3_^7^.

However, the latest members of the periodic table of elements were produced using an ion beam of doubly magic ^48^Ca. This particularly stable isotope of calcium (Ca, *Z* = 20) took on a central role in what has been undoubtedly the most successful method for the synthesis of latest SHEs^8^. But with californium (Cf, *Z* = 98) as the heaviest target element available in large enough amounts, the dream team of ^48^Ca and actinides has reached its limits: An annual production of einsteinium (Es, *Z* = 99), as the next logical target element for the synthesis of an element with *Z* = 119, yields only a few tens of micrograms of a radioisotope with a half-life of less than 300 days^9^. This quantity is about 300 times lower than what is required for a typical target (see **C** in Fig. 1). Although all actinide elements beyond uranium (U, *Z* = 92) generally originate from nuclear reactors, the heaviest ones rely on long-term irradiations with most intense thermal neutron fluxes in dedicated facilities, such the high-flux isotope reactor, HFIR, at Oak Ridge National Laboratory, USA or the SM-3 reactor in Dimitrovgrad, Russian Federation. The costly operation of these dedicated facilities, limitations in the lengthy production pathways themselves as well as the subsequent complex, large-scale radiochemical processing are the general reasons for the scarce availability of heavy actinides.

Otherwise limiting the production of SHEs is the current state of target technology. Ever growing ion beam intensities with newest ion sources and accelerators have left the target makers in the dust. Admittedly, improving today’s target technology above and beyond is not an easy task: While passing through the thin target structure of only a few micrometers, the ion beam not only considerably heats the material locally, but enhances diffusion of the present chemical elements and co-deposited compounds through the introduction of structural defects in the target itself. Therefore, the targets degrade over the long irradiation times by sputtering, evaporation as well as chemical reactions facilitated through heat and faster diffusion. The accepted, somewhat easy solution to that problem has been large rotating target assemblies, thus exposing individual segments only for a fraction of the time to the harmful conditions of a high-intensity ion beam. The downside, of course, is the larger amount of target material needed. Promising results regarding an improvement of the current technology have been achieved with intermetallic targets^10^, which represent a better alternative in comparison to targets prepared by molecular electroplating. However, considerable efforts are need before such a technology finds broad application in SHE synthesis experiments. Hence, in absence of better target alternatives, physicists have had to turn to heavier, but somewhat less favorable projectile isotopes, such as the above-mentioned ^50^Ti as well as ^51^V or ^54^Cr. Using these, and with Japanese scientists leading the way, the hunt for elements with atomic numbers *Z* = 119 and *Z* = 120 has only just begun^11^. Regardless, it remains to be seen if the increasingly short-lived radioisotopes of those elements of the 8^th^ period survive the transit time of roughly 1 μs through the adjacent separator to the detector (see **D** in Fig. 1) in these years-long irradiation experiments.

## Chemistry experiments with new elements

The law of periodicity, established by D. I. Mendeleev more than 150 years ago, in combination with sophisticated theoretical calculations based on relativistic density functional theory, help chemists to select proper chemical systems and devise suitable experiments for the investigation of SHEs. In order to optimize these setups for highest efficiencies and speed, a first technical problem to address is the transition from the physics instrumentation, such as a separator, into the self-contained chemistry experiment (see **E** in Fig. 1). Depending on the envisaged experiment, the provided interface must not exceed a thickness of a few micrometers in order for the SHEs not to get stuck inside as well as it may have to be stable to large pressure differences or intense heat loads. As the requirements vary for various selected chemical systems, an interface often needs to be largely reconstructed or even designed from scratch for individual experiments.

After the successful chemical characterization of copernicium (Cn, *Z* = 112) in its elemental state, chemists have performed several experiments with flerovium (Fl, *Z* = 114), likewise trying to preserve the elemental state. Whereas first experiments concluded on an unexpected higher volatility of Fl compared to Cn^12^, subsequent studies only partly supported the first findings^13^. Meanwhile, full relativistic quantum mechanical computations have consistently contradicted the very first investigation with Cn surpassing Fl in terms of their relative volatilities^14^. Open questions have remained on whether chemists fully managed to exclude a possible chemical compound formation. Conclusive results have yet to be presented with high enough measurement statistics (see **F** in Fig. 1). To achieve the latter in the light of extremely low production rates, such an experiment depends on costly month-long access to a dedicated accelerator facility. This direct access to an accelerator has proven furthermore vital during the preparation of new experiments, which need to be benchmarked using the lighter homologs of the targeted SHE. Only accelerator-based model experiments with short-lived species, produced in the same way as the SHEs, can serve as a truly reliable benchmark. Due to the highly ionized state of nuclear fusion products entering the chemistry experiment, high oxidation states of the element under investigation are feasible, thus possibly interfering with expectations based on the in-situ formation of typical chemical compounds as observed in offline experiments.

With the finalized, or far progressed chemical characterization of Cn and Fl, radiochemists can proceed in different direction in the periodic table. Moving forward, they face with nihonium through tennessine presumably comparably less volatile and chemically more reactive elements, and beyond flerovium only short-lived species with half-lives below one second (see **G** in Fig. 1). Looking backwards, three elements, namely meitnerium (Mt, *Z* = 109), darmstadtium (Ds, *Z* = 110), and roentgenium (Rg, *Z* = 111), have yet dodged a chemical assessment completely (see **H** in Fig. 1).

Moving in either direction demands for considerable improvements of existing experiments or entirely new approaches. Heading towards the heaviest elements requires much faster processing times in the millisecond-regime and the highest efficiencies as to the ever-decreasing production rates. In addition, a drastic improvement towards high-temperature alpha-detector technology is essential for efficiently identifying less volatile SHEs in gas adsorption chromatography experiments. Diamond- as well as SiC-based detectors have caught the attention of scientists with promising developments on their way^15^. Meanwhile the more stringent time constraints imposed by the short half-lives may be tackled by gas stopping cells. These have the capability to stop and extract ions of SHEs into an adjacent chemical apparatus within a few tens of milliseconds after emerging out of the separator^16^. Offering similarly fast processing times is vacuum adsorption chromatography^17^. In this technique, the undirected propagation of SHEs in an evacuated environment is overcompensated by high particle velocities.

On the other hand, turning to the so far uncharted territory of presumed noble metals Mt through Rg, radiochemists have to devise entirely new chemistry experiments. As suggested previously, an electrochemical approach might do the trick here for the coinage-metal homologs^18^. Although radioisotopes with long enough half-lives could be readily produced at much larger amounts compared to heaviest SHEs, these elements suffer so far on being outshone by their more prestigious colleagues at the upper end of the 7^th^ period. Due to their allegedly lower attractiveness, they are a tough act to follow during decision meetings on what to do next at a large accelerator. Nonetheless, as chemists increasingly reach physical limits with their heaviest study objects, Mt, Ds, and Rg might soon come back into the limelight.

Although not even produced, the discovery of yet another element would open up the 8^th^ period of the periodic table. Despite the questionable prospects regarding the survival probabilities of elements beyond Og past physical pre-separation (see above), those members of the 8^th^ period bring us closer to a new set of orbital, namely the *g*-orbitals. The presumably next elements to be discovered with atomic numbers *Z* = 119 and *Z* = 120 are predicted to be alkali and alkaline-earth metals eka-francium (8*s*^1^) and eka-radium (8*s*^2^)^19^. For the presumed alkali metal eka-Fr, one might expect a similar chemical behavior as for Nh of group 13 with its alkali-like behavior. The latter arises due to the strong relativistic stabilization of the 7*s*^2^-orbital. Thus, radioisotopes of eka-Fr with a certain longevity could be chemically studied in gas adsorption chromatography experiments such as currently under development for nihonium.

Further on, elements with atomic numbers 121–138 may be referred to as 5*g*-elements, even though an actual 5*g*-occupation in the atomic ground state becomes probable only for *Z* > 124^20^. If radiochemists ever get hold of those elements, they will be confronted with challenging chemistry: The similarly small binding energies of all electrons in the 8*s*, 8*p*_1/2_, 7*d*, 6*f*, and 5*g* shells suggest high oxidations states and orbital-mixing of unknown character^21^. Accurate theoretical prediction in this part of the periodic table are a particular challenge, which is why there are only very few studies available on potential chemical compounds. Regardless of the possibly exciting chemistry of many more elements up to a critical electronic limit at *Z* = 172^19^, nuclear stability likely puts an early end to these dreams.

## Outlook

If physicists succeed in the synthesis of an element with *Z* = 119 or 120, the hitherto unexplored *g*-orbitals come within reach. Unfortunately, as to the expected extremely short half-lives of produced species, it is highly questionable if any chemist will ever study these elements experimentally. Beyond that, the to-do list for radiochemists is manifold: On the way to study so far chemically unknown SHEs, challenges await, ranging from ion beam production and target stability towards new chemical experiments demanding for dedicated detector developments and the introduction of innovative instrumentation. With the gained insights, experimentalists provide valuable input for theoretical chemistry in the field of SHEs, as the emerging results from experimental studies can be used as benchmark for the advancement of predictive theory.
